# Vanishing pancreas: CT and MRI features and imaging diagnostic strategies

**DOI:** 10.1186/s13244-025-01998-4

**Published:** 2025-07-05

**Authors:** Yanjin Qin, Danyang Xu, Yuxin Wu, Xiaoqi Zhou, Chenyu Song, Zhi Dong, Lujie Li, Meicheng Chen, Yanji Luo, Huasong Cai, Mimi Tang, Shi-Ting Feng

**Affiliations:** Department of Radiology, The First Affiliated Hospital, Sun Yat-Sen University, Guangzhou, China

**Keywords:** Dorsal pancreatic agenesis, Intra-pancreatic fat deposition, Computed tomography, Magnetic resonance imaging

## Abstract

**Abstract:**

The vanishing pancreas is a frequently overlooked condition which can result from partial or complete dorsal pancreatic agenesis, intra-pancreatic fat deposition (IPFD) and pancreatic atrophy caused by chronic pancreatitis. A variety of diseases, including cystic fibrosis, maturity-onset diabetes of the young type 8, Shwachman-Diamond syndrome, and Johanson-Blizzard syndrome, can manifest as IPFD. Dorsal pancreatic agenesis can, albeit rarely, coexist with abnormalities or tumors. This review aimed to summarize the various causes that may result in partial or complete vanishing pancreas on computed tomography/magnetic resonance imaging (CT/MRI). We provide a comprehensive review of these imaging findings and their corresponding clinical characteristics, which are crucial for ensuring an accurate diagnosis.

**Critical relevance statement:**

By reviewing various causes of pancreatic vanishing, we summarize these imaging findings and their corresponding clinical characteristics, which is crucial for ensuring an accurate diagnosis and patient management.

**Key Points:**

- Imaging findings of partial or complete pancreatic vanishing reveal a hypodense pancreas (resembling fat density) or visibility of only the pancreatic head and proximal body.
- Pancreatic vanishing can result from dorsal pancreatic agenesis, intra-pancreatic fat deposition, and atrophy caused by chronic pancreatitis.
- Intra-pancreatic fat deposition is associated with genetic and systemic diseases.

**Graphical Abstract:**

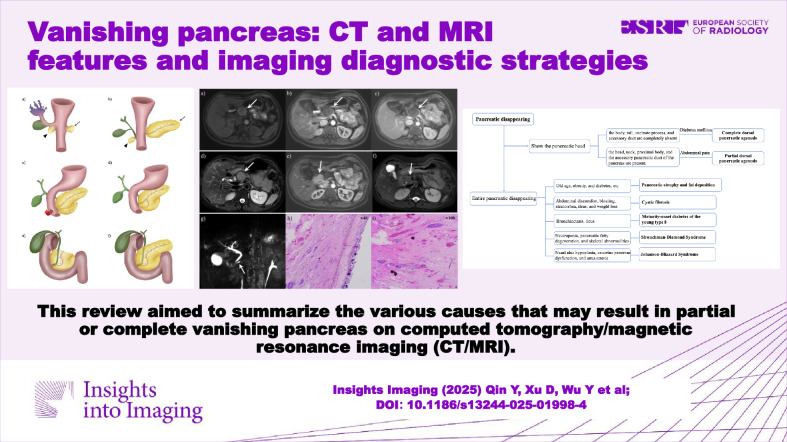

## Introduction

Pancreatic vanishing is a frequently overlooked condition characterized by the partial or complete absence of pancreatic tissue on imaging studies. This phenomenon can result from dorsal pancreatic agenesis [1], intra-pancreatic fat deposition (IPFD) and pancreatic atrophy secondary to chronic pancreatitis [2, 3]. The incidence of pancreatic vanishing is not well-documented. Computed tomography (CT) and magnetic resonance imaging (MRI) play a crucial role in diagnosing and differentiating these conditions. Understanding the imaging features and clinical implications of pancreatic vanishing is essential for accurate diagnosis and management. Misdiagnosis can lead to inappropriate treatment. In the present study, therefore, we summarize the characteristics of these diseases to aid in correct diagnosis and propose a differential diagnosis strategy.

### Pancreatic vanishing caused by dorsal pancreatic agenesis

#### Definition

Complete dorsal pancreatic agenesis, a rare and fatal disease, has been exceptionally infrequently documented in medical literature. In contrast, partial dorsal pancreatic agenesis, first noted as an autopsy finding in 1911, remains an extremely unusual congenital abnormality. Approximately 100 cases have been reported to date [1, 4]. The pancreatic body, tail, and minor papilla as well as the accessory duct are absent in cases of complete dorsal agenesis, while the neck and proximal body of the pancreas, as well as the remnants of the small papilla and accessory pancreatic duct are present in cases of partial dorsal agenesis [5, 6].

#### Etiology

**Fig. 1:**
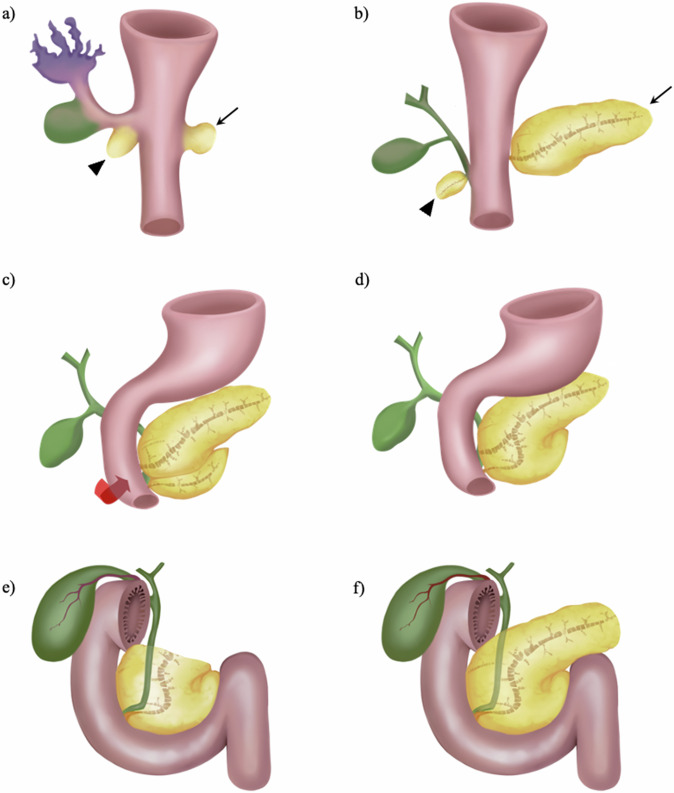
Drawings depict the typical embryological development of the pancreas. The ventral pancreatic bud (marked with arrowheads in **a** and **b**) originates from the hepatic diverticulum, while the dorsal pancreatic bud (indicated by arrows in **a** and **b**) emerges from the dorsal mesogastrium. **c** Duodenal expansion induces the ventral pancreatic bud to rotate, pass posterior to the duodenum from right to left, and fuse with the dorsal pancreatic bud. **d** Ultimately, the ducts of both the ventral and dorsal pancreas unite, with the majority of pancreatic secretions being drained through the ventral duct, completing the developmental process. **e** Complete form of dorsal pancreatic agenesis. **f** Partial form of dorsal pancreatic agenesis

At the end of the fourth embryonic week, endoderm cells proliferate adjacent to the caudal margin of the liver diverticulum, giving rise to ventral pancreatic buds, while contralateral cells underwent proliferation and formed dorsal pancreatic buds (Fig. 1a–d) [7, 8]. The lower head and the uncinate process of the pancreas develop from the ventral buds, whereas the upper part, isthmus, body, and tail of the pancreas are generated by dorsal buds [6, 9]. Developmental failures due to aberrant embryogenesis may lead to partial or complete agenesis of the dorsal pancreas (Fig. 1e, f). The exact genetic pathogenesis of dorsal pancreatic agenesis remains unknown. Several studies have shown that mutations in specific genes may lead to dorsal pancreatic agenesis, including GATA binding protein 6 (GATA6) and hepatocyte nuclear factor 1B (HNF1B) [10]. Besides, mutations in two genes, pancreas transcription factor 1 alpha (PTF1A) and pancreatic duodenal homeobox 1 (PDX1), lead to dorsal pancreatic agenesis in mice [11, 12].

#### Clinical symptoms

Most patients with dorsal pancreatic agenesis remain asymptomatic. When symptoms do occur, abdominal pain is more frequently reported in cases of partial dorsal pancreatic agenesis, whereas diabetes mellitus is commonly observed in those with complete dorsal pancreatic agenesis [13, 14].

#### Imaging findings

**Fig. 2:**
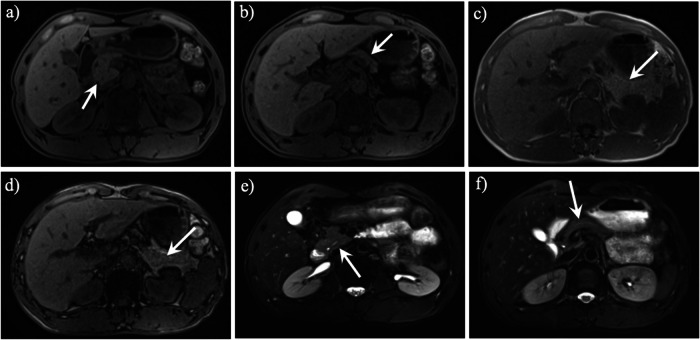
A 48-year-old man with partial dorsal pancreatic agenesis. Axial fat-suppressed T1-weighted MR images (**a**, **b**) revealed a normal-appearing pancreatic uncinate process, head (**a**, arrow), neck (**b**, arrow), with the body-tail portion partially absent. The in-phase (**c**) and out-of-phase (**d**) T1-weighted MR images revealed that the partial absence of the pancreatic body-tail portion was filled by the adjacent retroperitoneal fat (arrow). Axial T2-weighted MR images (**e**, **f**) showed only the pancreatic duct in the head, neck and partial body of the pancreas with linear high signal (arrow), but the pancreatic duct of the tail was invisible

**Fig. 3:**
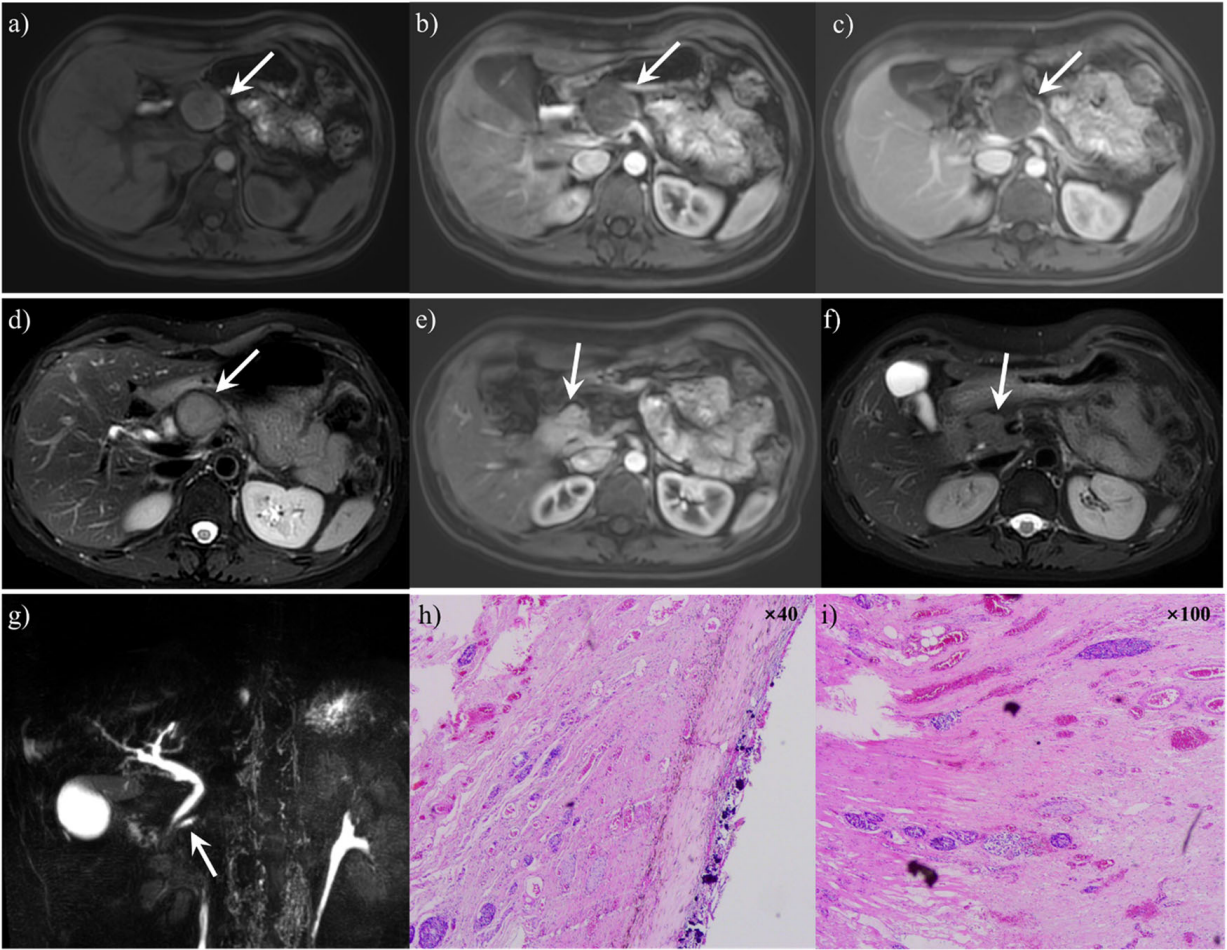
A 42-year-old woman presented with dorsal pancreatic agenesis and a pancreatic pseudocyst. **a** Axial T1-weighted fat-suppressed MR image revealed a well-defined mass (arrow) with slightly hyperintensity and peripheral hyperintensity. Arterial (**b**) and venous (**c**) phase MR images showed that the tumor had no significant enhancement. The tumor (arrow) showed a slightly hyperintense signal on axial T2-weighted MR image (**d**). Arterial phase MR image (**e**) and T2-weighted MR image (**f**) showed that only the head of the pancreas was visible (arrow). Coronal Magnetic Resonance Cholangiopancreatography (MRCP) (**g**) showed that the main pancreatic duct (arrow) was short and the pancreatic duct in the body and tail of the pancreas was not visualized. **h**, **i** Pathology confirmed a pancreatic pseudocyst, though post-tumor change after complete regression could not be excluded

Abdominal CT and MR imaging of patients with complete dorsal pancreatic agenesis reveal the presence of only the pancreatic head, with the body, tail, and accessory duct completely absent. In contrast, individuals with partial dorsal pancreatic agenesis exhibit the head, neck, and proximal body of the pancreas, as well as remnants of the accessory pancreatic duct (Fig. 2). On magnetic resonance cholangiopancreatography (MRCP), the dorsal pancreatic duct could not be fully visualized (Fig. 3). Three key features should raise suspicion: (1) unpaired ventral pancreatic configuration terminating at the superior mesenteric artery (SMA) margin, (2) absent dorsal parenchyma beyond the SMA plane, and (3) lack of the normal ductal “herringbone” pattern [15, 16]. These findings help differentiate it from pancreatic atrophy (preserved ductal architecture), chronic pancreatitis (calcifications or ductal strictures), or post-pancreatectomy states (surgical clips or anastomoses).

#### Coexisting with other entities or differential diagnostic

**Fig. 4:**
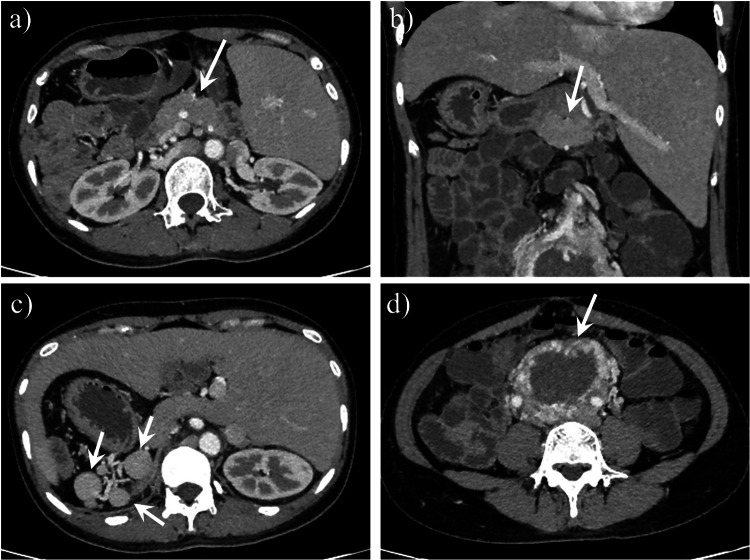
A 35-year-old woman had dorsal pancreatic agenesis, polysplenia syndrome, total situs inversus viscerum and retroperitoneal paraganglioma. Axial (**a**) and coronal (**b**) contrast-enhanced computed tomography (CT) scans showed the pancreatic head and neck (arrow) and the absence of the pancreatic body-tail portion. Axial contrast-enhanced CT scan (**c**) showed multiple accessory spleens in the right upper quadrant (arrow), and most of the liver was located in the left upper abdomen. Axial contrast-enhanced CT scan (**d**) showed a markedly inhomogeneous enhanced retroperitoneal mass (arrow), which was pathologically confirmed as a paraganglioma

**Fig. 5:**
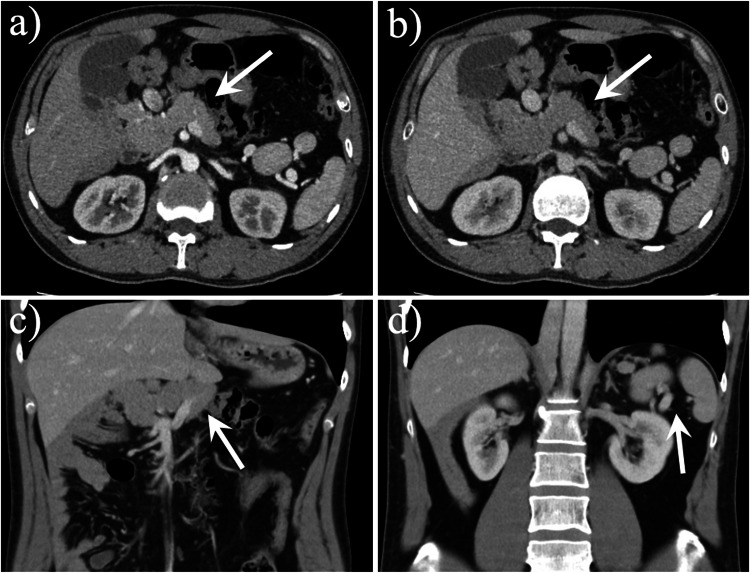
A 32-year-old man with dorsal pancreatic agenesis associated with polysplenia syndrome. Axial contrast-enhanced computed tomography (CT) images (**a**, **b**) and coronal contrast-enhanced CT image (**c**) showed the pancreatic head (arrow) and neck and the absence of part of the pancreatic body-tail portion. Coronal contrast-enhanced CT image (**d**) showed multiple accessory spleens in the left upper quadrant (arrow)

**Fig. 6:**
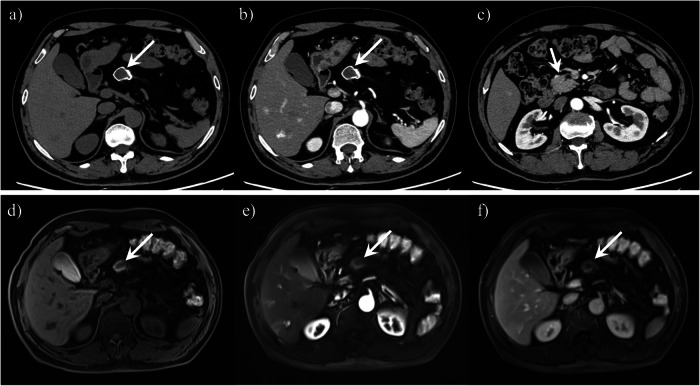
A 62-year-old man presented with dorsal pancreatic agenesis and a pancreatic pseudocyst. Axial unenhanced computed tomography (CT) image (**a**) showed that the well-defined lesion (arrow) was slightly hypodense with peripheral calcification. Contrast-enhanced CT image (**b**) showed that the tumor had no enhancement (arrow). Contrast-enhanced CT image (**c**) showed only the pancreatic uncinate process and head (arrow), but most of the rest of the pancreas was not visualized. The tumor (arrow) showed slightly hypointensity with peripheral hyperintensity on axial T1-weighted fat-suppressed MR image (**d**). Arterial (**e**) and venous (**f**) phase MR images showed that the tumor had no enhancement

**Fig. 7:**
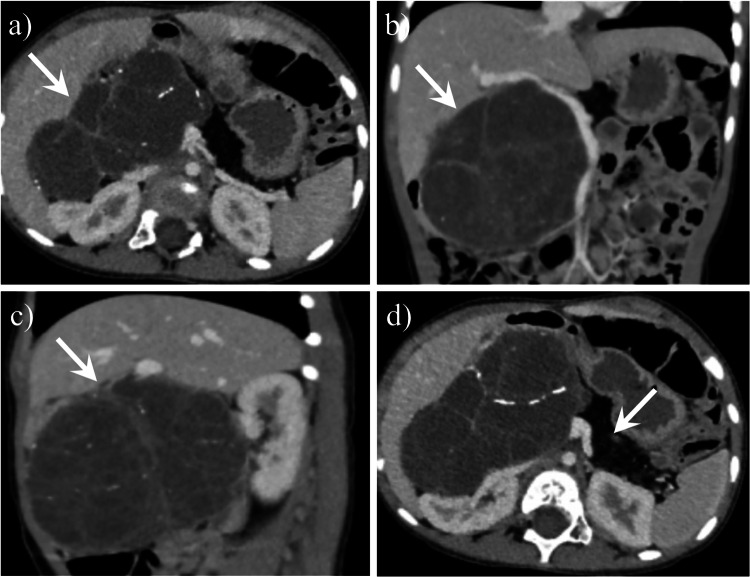
A 2-year-old girl with dorsal pancreatic agenesis coexisted with serous cystadenoma. Axial (**a**), coronal (**b**) and sagittal (**c**) contrast-enhanced computed tomography (CT) images revealed a large right intraperitoneal mass (arrow) with enhancing septage and central stellate calcification. Axial CT image (**d**) revealed the “absence” of the pancreatic body-tail portion, and the corresponding region showed fat density (arrow). This is the second case of a serous cystadenoma of the pancreas occurring in the context of dorsal pancreatic agenesis

**Fig. 8:**
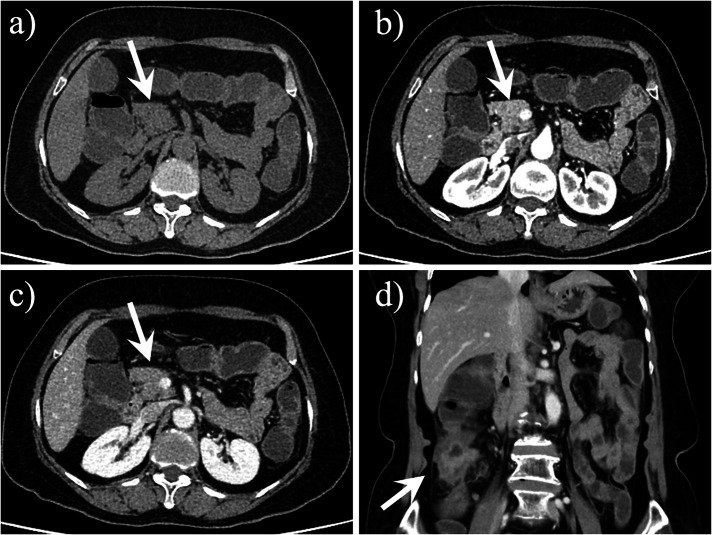
A 76-year-old woman with dorsal pancreatic agenesis coexisted with colon cancer. Axial unenhanced computed tomography (CT) image (**a**) and contrast-enhanced CT images (**b**, **c**) showed the pancreatic head (arrow) and the absence of the pancreatic body-tail portion. Coronal contrast-enhanced CT image (**d**) showed marked irregular wall thickening in the proximal ascending colon‌ (arrow), with postoperative pathological examination confirming the diagnosis as colorectal adenocarcinoma‌

Other abnormalities may coexist with dorsal pancreatic agenesis such as polysplenia syndrome (Figs. 4, 5) [17–20] or ectopic spleen [21], heterotaxy syndrome [19], bowel malrotation [22, 23], pseudocyst (Figs. 3, 6), choledochal cyst [24, 25], annular pancreas [23, 26], vaginal atresia [27], and cardiac [19] or vascular [19, 23, 26, 28] abnormalities. Additionally, the coexistence of pancreatic tumors and dorsal pancreas agenesis has been reported. These tumors include malignant intraductal papillary mucinous neoplasm (IPMN) [29], solid pseudopapillary tumors [30, 31], adenocarcinomas [26, 32], neuroendocrine tumor (NET) [33], and serous cystadenoma of the pancreas (Fig. 7) [34]. Among tumors coexistence of dorsal pancreas agenesis, solid pseudopapillary tumor is the most common tumor. In this review, we also presented an interesting case of dorsal pancreatic agenesis coexisting with colorectal adenocarcinoma (Fig. 8).

Regarding the coexistence of pancreatic tumors and dorsal pancreatic agenesis, there are some questions worth considering:

1. Is the incidence of dorsal pancreatic agenesis with tumors higher than that of normal pancreas with tumors? A few case reports have described the coexistence of dorsal pancreatic agenesis with pancreatic tumors, such as serous cystadenoma and neuroendocrine tumors [33, 34]. These cases suggest that dorsal pancreatic agenesis may create a unique microenvironment that could influence tumor development, but larger studies are needed to confirm this observation.
2. Is dorsal pancreatic agenesis in coexistence with tumors an incidental phenomenon or is there an intrinsic link between dorsal pancreatic agenesis and pancreatic tumors? Whether the coexistence of dorsal pancreatic agenesis and pancreatic tumors is incidental or reflects an intrinsic link is still debated. Some authors hypothesize that the absence of the dorsal pancreas may alter pancreatic physiology, such as ductal drainage and parenchymal stress, potentially creating conditions that favor tumorigenesis [35]. However, this remains speculative due to the rarity of reported cases.
3. If there’s a link, what’s the mechanism by which dorsal pancreatic agenesis predisposes to tumors or inhibits tumor growth? The mechanism underlying the association between pancreatic tumors and dorsal pancreatic agenesis remains uncertain. However, some theories suggest that dorsal pancreatic agenesis may increase the risk of chronic pancreatitis, which itself is a well-established risk factor for pancreatic tumors [36].

Research that addresses these questions requires a great deal of evidence to support it, and these research studies will be valuable and interesting in the future.

### Pancreatic vanishing caused by fatty pancreas disease

#### Definitions

Fatty pancreas disease, with a prevalence between 16% and 35%, refers to the fat deposition in the pancreas [37, 38]. A dozen terms, such as ‘intra-pancreatic fat deposition’ [39], ‘pancreatic steatosis’ [40], ‘fatty infiltration of the pancreas’ [41], ‘pancreatic fat accumulation’ [42], and ‘non-alcoholic fatty pancreas’ [43], have been used to refer to this phenomenon. However, a consensus on the best terms has not been reached worldwide. In this review, we used the term ‘intra-pancreatic fat deposition (IPFD)’ to manifest as fatty change within the pancreas [39].

#### Etiology

There are two distinct mechanisms that will lead to fatty change within the pancreas: fatty replacement and fatty infiltration. Fatty replacement involves the death of pancreatic acinar cells and their subsequent replacement by adipocytes, leading to a loss of functional pancreatic tissue [44]. In contrast, fatty infiltration is characterized by the accumulation of fat within the pancreas without significant cell death [45]. IPFD can include intra-lobular fat (for example, islet cells or acinar cells) or inter-lobular fat [45]. Risk factors such as older age, obesity, alcohol, diabetes, enlarged abdominal waist circumference, and metabolic variables (including glycated hemoglobin, lipid levels, and systolic blood pressure) have been linked to the occurrence of IPFD [45, 46]. Additionally, congenital diseases that can manifest as IPFD are as follows:

Cystic fibrosis is an autosomal recessive disorder that affects the exocrine function of multiple organs, including the lungs, liver, gastrointestinal tract, pancreas, sweat glands, and urogenital system. It arises from genetic variations in the CFTR gene [47]. It primarily affects the lungs and manifests as bronchiectasis. In classic cystic fibrosis, secretions become thicker, leading to pancreatic autodigestion and replacement of the pancreatic tissue with fat. Notably, nearly 80% of individuals with cystic fibrosis suffer from pancreatic insufficiency, which typically manifests as abdominal discomfort, bloating, steatorrhea, ileus, and weight loss [48].

Maturity-onset diabetes of the young type 8 (MODY8) is a monogenic inherited disorder and exhibits as both endocrine and exocrine pancreatic dysfunction [49]. Patients often exhibit exocrine pancreas dysfunction in childhood, progressing to diabetes in adulthood [50].

Shwachman-Diamond syndrome (SDS) is a rare autosomal recessive disorder [51], characterized by neutropenia, IPFD, and metaphyseal dysplasia [52, 53].

Johanson-Blizzard Syndrome (JBS) is an exceptionally rare autosomal recessive disorder caused by UBR1 gene mutations [54], featuring congenital exocrine pancreatic insufficiency, nasal wings hypoplasia or aplasia, or other malformations [55–57].

#### Clinical symptoms

Patients with IPFD present with symptoms associated with loss of pancreatic function. These symptoms include metabolic syndrome (hypertension, low plasma HDL cholesterol levels, hypertriglyceridemia, impaired glucose regulation and abdominal obesity), dyspepsia, chronic diarrhea, steatorrhea, and signs of diabetes due to low insulin secretion [45].

#### Imaging findings

**Fig. 9:**
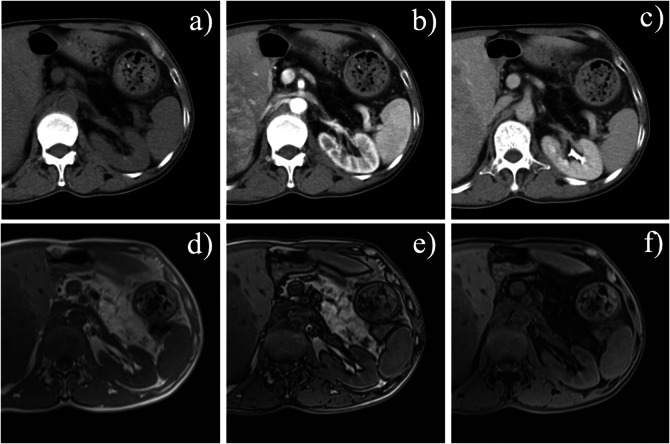
A 50-year-old man with intra-pancreatic fat deposition coexisted with hepatocellular carcinoma. **a**–**c** Axial unenhanced and contrast-enhanced computed tomography (CT) images showed fat density in the pancreatic area without enhancement. Axial in-phase (**d**) and out-of-phase (**e**) T1-weighted MR images showed hyperintensity signal in the pancreatic region, and this region was hypointense on T1-weighted fat-suppressed MR image (**f**). At the same time, part of the liver tumor can be seen in these images.

On CT scan, the pancreas has hypodense parenchyma in patients with IPFD. The pancreas shows similar density or signal to the surrounding retroperitoneal fat. T1-weighted MR image of the pancreas shows diffuse and marked hyperintensity of pancreas, with residual hypointense reticulation and contours, indicating the residual stroma (Fig. 9). The superior mesenteric vessels and splenic vein are clearly visualized. The ducts remain preserved in IPFD, which is a critical point for differentiating it from dorsal pancreatic agenesis.

#### Coexisting with other entities or differential diagnostic

**Fig. 10:**
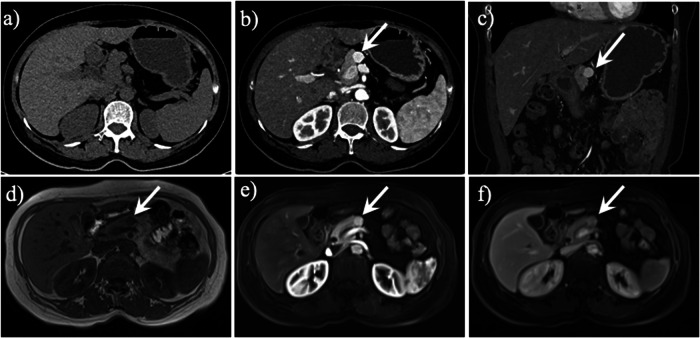
A 62-year-old woman with intra-pancreatic fat deposition coexisted with a pancreatic neuroendocrine tumor. Axial unenhanced and contrast-enhanced computed tomography (CT) images (**a**, **b**) and coronal contrast-enhanced CT image (**c**) showed only the head of the pancreas (arrow), while the pancreatic body-tail portion showed fat density. There was an obvious homogeneous hypervascular nodule in the neck of the pancreas (arrow), which was confirmed as a neuroendocrine tumor by postoperative pathology. Axial T1-weighted non-contrast (**d**), arterial phase (**e**), and venous phase (**f**) images demonstrated ‌fat-equivalent signals‌ in the pancreatic body and tail‌ region. A ‌nodule in the pancreatic neck‌ exhibits ‌mild hypointensity‌ on non-contrast T1WI ‌(arrow) ‌, with ‌marked arterial phase enhancement ‌(arrow) and ‌venous phase enhancement intensity‌ comparable to that of the pancreatic head parenchyma (arrow)

IPFD is associated with several prevalent clinical conditions, including metabolic syndrome, type 2 diabetes mellitus (T2DM), cardiovascular risk, acute and chronic pancreatitis, pancreatic fibrosis, pancreatic neuroendocrine tumors, and pancreatic cancer [58]. Here, we presented a case of IPFD coexisting with pancreatic neuroendocrine tumors (Fig. 10).

### Pancreatic vanishing caused by chronic pancreatitis

#### Definition

IPFD is also a manifestation of advanced stages of chronic pancreatitis caused by pancreatic atrophy. Chronic pancreatitis (CP) is marked by the progressive fibrosis of the pancreas, leading to exocrine and endocrine insufficiency [59].

#### Etiology

CP is a multifactorial disease with diverse etiologies. The most significant risk factor for CP in adults is alcoholism [60]. In children, the primary causes include genetic diseases (particularly cystic fibrosis) and anatomic abnormalities [60]. Other risk factors include toxic-metabolic factors (such as alcohol consumption, chronic renal failure, and hypercalcemia), idiopathic factors (such as early and late onset), genetic factors (e.g., autosomal dominant and autosomal recessive modifier genes), and autoimmune pancreatitis, among others [61]. The main pathology of “atrophy parenchyma” involves the progressive loss of pancreatic parenchyma and fibrosis due to chronic inflammation.

#### Clinical symptoms

Patients with CP present with severe abdominal pain, exocrine pancreatic insufficiency (manifesting as steatorrhea, weight loss, and malnutrition), and diabetes [59].

#### Imaging findings

**Fig. 11:**
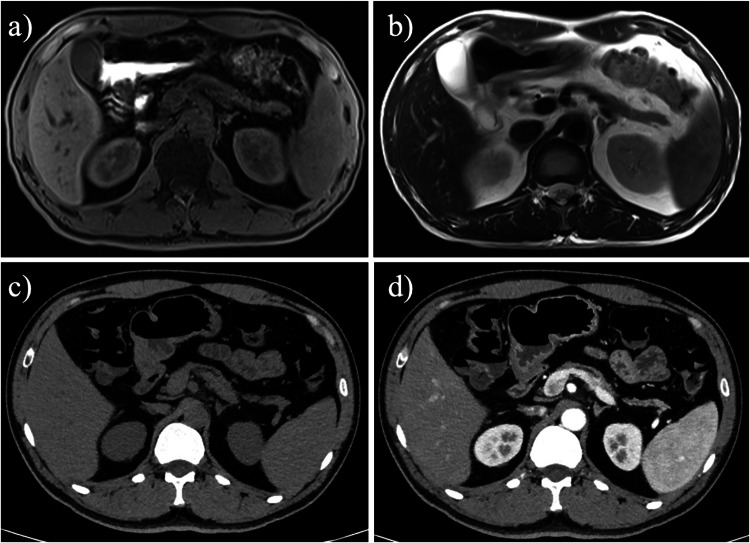
A 33-year-old man with chronic pancreatitis and pancreatic atrophy. Axial T1-weighted fat-suppressed MR image (**a**), T2-weighted MR image (**b**), axial unenhanced computed tomography (CT) image (**c**), and contrast-enhanced CT image (**d**) demonstrated thread-like pancreatic morphology with marked volume reduction

The most common imaging findings of CP on CT and MRI scans are pancreatic duct dilation and pancreatic atrophy (Fig. 11) [62]. Additionally, pancreatic calcifications and pseudocysts can be observed on CT. MRCP can clearly display pancreatic duct strictures or abnormal dilation and provide quantitative measurements of exocrine function [63].

#### Coexisting with other entities or differential diagnostic

Obstructive chronic pancreatitis, with diffuse ductal dilatation and diffuse pancreatic parenchymal atrophy, can mimic pancreatic ductal adenocarcinoma at imaging. The duct penetrating sign, the presence of biliary or main pancreatic duct skip strictures may help to diagnose chronic pancreatitis. An abrupt cutoff of the main pancreatic duct in the pancreatic head suggests a malignant stricture. A serum CA19-9 level greater than 40 U/mL is 90% specific and 81% sensitive for a pancreatic malignancy [64].

## Conclusion

Pancreatic vanishing is demonstrated by pancreatic agenesis, IPFD and pancreatic atrophy. In patients with dorsal pancreatic agenesis, only part of the pancreas is visible. Additionally, in patients with IPFD, the entire pancreas exhibits fatty density/signal. Pancreatic vanishing can sometimes be overlooked, but it is also important for diagnosis at times. Therefore, our study highlights the essential points for diagnosing these pancreatic diseases, taking into account their clinical and imaging features. As described in this article, a systems-based approach for addressing potential complications provides a strong interpretive framework for reviewing postoperative CT or MRI examinations. By following this systems-based approach, we aim to achieve a clearer categorization of diseases associated with the “pancreatic vanishing.”

## Data Availability

The datasets generated and analyzed during the current study are not publicly available because they contain sensitive patient information, but are available from the corresponding author upon reasonable request.

## Abbreviations

CT: Computed tomography
GATA6: GATA binding protein 6
IPFD: Intra-pancreatic fat deposition
MODY-8: Maturity-onset diabetes of the young type 8
MRCP: Magnetic resonance cholangiopancreatography
MRI: Magnetic resonance imaging
